# Sensitive detection of tumour cells in effusions by combining cytology and fluorescence *in situ* hybridisation (FISH)

**DOI:** 10.1038/sj.bjc.6601942

**Published:** 2004-06-29

**Authors:** M Fiegl, A Massoner, M Haun, W Sturm, H Kaufmann, R Hack, J Krugmann, M Fritzer-Szekeres, K Grünewald, G Gastl

**Affiliations:** 1 Division of Haematology and Oncology, Department of Internal Medicine, Medical University of Innsbruck, Innsbruck, Austria; 2 Division of General Internal Medicine, Department of Internal Medicine, Medical University of Innsbruck, Innsbruck, Austria; 3 Department of Internal Medicine I, Division of Oncology, Medical University of Vienna, Vienna, Austria; 4 Department of Pulmonology, Krankenhaus Natters, Natters/Tyrol, Austria; 5 Institute of Pathology, Medical University of Innsbruck, Innsbruck, Austria; 6 Clinical Institute of Medical and Chemical Laboratory Diagnostics, Medical University of Vienna, Vienna, Austria

**Keywords:** malignant effusion, FISH, cytology, aneuploidy, transudate, molecular staging

## Abstract

Diagnosis of malignant cells in effusions is important for staging procedures and resulting therapeutic decisions. Cytodiagnostics in effusions is sometimes difficult since reactive mesothelial cells can mimic malignant cells. We used fluorescence *in situ* hybridisation (FISH) in single-colour or if appropriate in dual-colour evaluation to detect chromosomal aberrations in effusion cells as markers of malignancy, to raise the diagnostic yield. Cytologic and FISH evaluations – by using probes representing several chromosomes always including chromosomes 11 and 17 – were performed in 358 effusion fluids. Cytology was positive for malignancy in 44.4% of all effusions, whereas FISH was positive in 53.9% (*P*=0.0001). The combination of cytology and FISH was diagnostic for malignancy in 60.9% of effusions. Diagnostic superiority of FISH was demonstrated in effusions from breast cancer, lung cancer, pancreatic cancer, and in effusions from the entire group of gynaecological and gastrointestinal carcinomas. In transudates (effusion protein <2.5 g dl^−1^), malignant cells were detectable by cytology, FISH, and combined use of both methods in 18.6, 30, and 37.1% of effusions, respectively, suggesting that cytologic and molecular analysis should be performed also with transudates. In conclusion, FISH in combination with conventional cytology is a highly sensitive and specific diagnostic tool for detecting malignant cells in effusions.

Effusion fluids in cancer patients can arise in the pleural, pericardial, and peritoneal space. Pathologic accumulation of such fluid is driven by different forces, among which are ‘paraneoplastic’ effects triggered directly or indirectly by tumour cells. For example, vascular endothelial growth factor (VEGF) can be released from tumour cells and it increases the permeability of peritoneal microvessels and thus contributes to effusion accumulation. On the other hand, removal of VEGF from the peritoneal cavity has been shown to inhibit ascites formation in ovarian cancer models (Byrne *et al*, 2003). About 10–15% of all effusions submitted for pathological analysis are tumour-associated (Runyon, 1994; Light, 2002), and about 50% of patients develop an effusion at some time during the course of disease. Clinically, effusion in tumour disease heralds local disease progression, relapse or metastasis (Raju and Kardinal, 1981). However, pleural or ascitic effusions do not always indicate advanced or metastatic malignant disease, with major clinical consequences. For example, in lung cancer, a cytologically negative effusion at primary diagnostic workup usually corresponds with a limited disease stage, whereas a cytologically positive pleural effusion represents a UICC/AJCC TNM stage T4 (TNM classification, 1997), translating into inoperable stage IIIB or IV disease. It is well documented that the quality of cytodiagnostics regarding tumour-associated effusions primarily depends on the investigator's experience to discriminate malignant from reactive effusions. The diagnosis of malignancy in effusions can be troublesome due to the cellular composition of effusions. The occurrence of single or clustered ‘activated mesothelial cells’ with morphological features of tumour cells can mimic malignancy (Koss, 1992). On the other hand, in effusions tumour cells may appear quite similar to normal cells, for example, small-cell lung cancer cells and lymphocytes (Chhieng *et al*, 2001). Due to these difficulties, cytopathologists traditionally adopt a rather cautious approach in the diagnosis of malignancy in effusions. In fact, the sensitivity for the cytological diagnosis of malignant cells in effusions even in patients with *known* cancer is astonishingly low, that is, about 50% (reviewed in Fiegl *et al*, 2003). Moreover, the specificity of cytological analysis in effusions does not reach 100%. Falsely positive results in effusions may occur due to chronic irritating states of different kinds (Kutty *et al*, 1981; Guzman *et al*, 1992). Thus, new diagnostic approaches are warranted to enhance the sensitivity and specificity of tumour cell detection in effusions. We and others used fluorescence *in situ* hybridisation (FISH) to sensitively detect tumour cells regularly characterised by numeric chromosomal aberrations (Fiegl *et al*, 2000, 2004; van Oostenbrugge *et al*, 2000). By identification of tumour-associated aneuploidy, FISH analysis has been successfully applied in tumour aspirates, urine, cerebrospinal fluid, cervical smears, sputum, and effusions (Cajulis *et al*, 1994; Chen *et al*, 1995; Ichikawa *et al*, 1996; Schenk *et al*, 1997; Mian *et al*, 1999; Fiegl *et al*, 2000, 2004; van Oostenbrugge *et al*, 2000).

The aims of this study were (1) to determine the percentage of effusions, which is the first manifestation of malignant disease and of relapse, (2) to test the diagnostic power of FISH as compared to cytology in effusions taken from different tumour entities, and (3) to compare the sensitivity of cytology and FISH in transudates. The study presented here includes an updated series of 358 effusion specimens, of which various cytological and molecular aspects have already been previously published (Fiegl *et al*, 2000, 2004).

## MATERIALS AND METHODS

### Effusion samples

In all, 403 effusion specimens were collected consecutively from 301 patients with various carcinomas. A total of 110 effusions originated from patients who had two or three repeated effusion punctures. Further, 45 of the effusion samples were excluded from analysis since the time interval between sample collections in an individual patient was less than 1 month, and, therefore, a significant alteration of cellular composition could not be expected. Thus, a total of 358 effusion fluids (198 of pleural, 153 of ascitic origin, five lavage, and two cyst fluids) were subjected to FISH analysis using centromeric probes for 2–6 chromosomes. An aliquot of 20–50 ml of each effusion was submitted to the Department of Pathology for cytological evaluation after routine staining (Giemsa, Papanicolau, or H&E). The first 201 effusions (series 1; Fiegl *et al*, 2000) were analysed at the University of Vienna Medical School between 1994 and 1998, whereas the remaining 157 effusions (series 2; Fiegl *et al*, 2004) were analysed at the University of Innsbruck Medical School between 2001 and 2002. For the definition of cutoffs for malignancy by FISH analysis (Table 2), cells from 15 and 66 control effusions, derived from patients with diseases other than cancer, were used in series 1 and 2, respectively.

A detailed description of the progeny of effusions, their underlying malignancies, and the proportion of effusions occurring as the first manifestation of malignant disease or of relapse is given in Table 1

**Table 1:** Origin of effusions, underlying malignancies and number of effusions representing first manifestation of disease or of relapse

				**Effusion**	
**Tumour entity**	**Patients (*n*)**	**Effusions (*n*)**	**A/P/L/C^a^**	**First manifestation of disease**	**First manifestation of relapse**
Breast	77	104	28/75/0/1	4	24
Lung	60	71	7/64	21	7
Pancreatic	33	34	31/3	1	1
Ovarian	26	29	18/9/1/1	10	2
Hepatoma	17	17	15/2		
UPC^a^	13	17	14/3	5	
Gastric	12	14	13/1	2	1
Colorectal	11	11	7/4		
Endometrial	9	11	4/3/4	1	4
Cholangio-cellular	8	9	9/0	1	
Renal	8	9	4/5		2
Haematological	9	10	2/8		
Mesothelioma	3	4	1/3	1	
Cervical	3	3	2/1	0	1
Others	12	15	1/14		2
Sum	301	358	156/195/5/2	46	44

a A=ascites; P=pleural effusion; L=lavage fluid; C=cyst fluid; UPC=unknown primary cancer.

. All patients had former or present histopathologically or cytologically verified tumours, mostly with distant metastasis or locally advanced, inoperable disease.

### Fluorescence *in situ* hybridisation analysis

Cells of at least 350 ml of effusion fluid (exception: lavages, cyst fluids, and a few effusions with 2–100 ml obtained) were gained by centrifugation, and, in case of macroscopic blood contamination, subjected to density gradient separation over Ficoll–Hypaque (Sigma, St Louis, MO, USA). Pelleted effusion cells were washed in phosphate-buffered saline, fixed in methanol–acetic acid (3 : 1, v v^−1^) and stored at −80°C. Directly fluorescence-labelled alpha-satellite DNA probes (SpectrumGreen ([excitation peak of 497 nm, emission peak of 524 nm) and SpectrumOrange (559/588 nm); Vysis Inc, Downers Grove, IL, USA) were applied in dual-colour FISH experiments. The probes used in this study were specific for the centromeres of chromosomes 7, 8, 11, 12, 17, and 18 for the first 201 consecutive effusions (Fiegl *et al*, 2000); subsequently, only probes representing chromosomes 11 and 17 were used (Fiegl *et al*, 2004). The standard protocol followed has been described in a previous report, with minor modifications (Drach *et al*, 1995).

### Fluorescence microscopy and definition of cutoffs for aneuploidy

A fluorescence microscope with × 60 and × 100 planar objectives and appropriate filter sets was used for FISH signal evaluation and documentation. All effusion cells in a field except for polynucleated granulocytes, which are easily distinguishable by nuclear shape, were analysed. The stringent criteria of FISH signal assessment were applied to avoid overestimation of hyperdiploidy, which may result from cellular and technical factors (reviewed in Eastmond *et al*, 1995). Signal counting was performed by two investigators, and intraobserver and interobserver counting variations were evaluated repeatedly. In order to evaluate the frequencies of aneusomic effusion cells with statistical reliability, centromeric signals of 100–1000 nuclei were scored, with high-number cell counting in samples with a low frequency of aneuploidy (Kibbelaar *et al*, 1993). When necessary, we used a two-tiered scoring procedure: (1) in all 358 effusions, scoring of nuclei in *single-colour FISH evaluation* was performed, and if aneusomy above cutoff for any of the tested chromosomes (Table 2

**Table 2:** Definition of criteria which enabled the diagnosis of tumour-associated aneuploidy by FISH^a^ in a two-step microscopic evaluation of effusion specimens

		**Step of evaluation**		
		**First: single-colour FISH**	**Second: dual-colour FISH**	**Specificity**
Effusions (*n*=358)	Series 1 (*n*=201)	*Malignant*, if above the respective cutoff (see Table 3)	*Malignant*, if ⩾20 cells with chromosomal gain detected, of which >50% aneuploid	100% (based on 15 control effusions)
	Series 2 (*n*=157)	*Malignant*, if ⩾5% of scored nuclei with chromosomal gain, or ⩾15% monosomic	*Malignant*, if ⩾20 cells with chromosomal gain detected, of which >60% aneuploid	97% (based on 66 control effusions)

a Between series 1 and series 2, the cutoffs in single-colour and dual-colour FISH evaluation differed, as indicated in the table. Briefly, in the *first step of evaluation*, nuclei in an effusion were evaluated in single-colour evaluation, and, if the percentage of nuclei was above cutoff for any of the evaluated chromosomes, a diagnosis positive for malignant cell involvement was given. If the diagnosis was negative, the *second step of evaluation* in dual-colour FISH evaluation followed: upon screening of about 10 000–20 000 nuclei, only nuclei with hyperdisomy were recorded; those with concordant signal gain (e.g., 4/4-pattern) were classified as polyploid, whereas those with discordant signal gain (e.g., 3/5-pattern) were classified as aneuploid. Aneuploidy above cutoff as indicated in the table was diagnostic for malignancy (also detailed in Fiegl *et al*, 2000, 2004).

) was present, malignancy could be documented; otherwise, in step (2), scoring of selected, namely hyperdisomic, nuclei was performed in *dual-colour FISH evaluation*, which allowed for the detection of rare FISH-aneuploid cells (Fiegl *et al*, 1999). For the analysis of the first 201 effusions (series 1) and the remaining 157 effusions (series 2), slightly different criteria to discriminate malignant from reactive effusions by FISH were used. This was necessary since the first author changed laboratories in 1998 and, therefore, cutoffs for background aneusomy and true, tumour-associated aneuploidy had to be redefined. In Table 2, the criteria which allowed diagnosis of malignant cell involvement in effusion series 1 and 2 are presented, and the diagnostic specificities as determined in control effusions are indicated. In series 1, cutoffs (Table 3

**Table 3:** Cutoff threshold levels for background aneusomy (percentage) for the six chromosomes examined in series 1 (1994–1998)

	**Signal number category**			
	**1**	**3**	**4**	**>4**
Chromosome 7	11.5	0.6	0.7	0.2
Chromosome 8	8.7	0.5	0.9	0.1
Chromosome 11	11.2	0.4	1.2	0.2
Chromosome 12	10.0	1.4	1.0	0.2
Chromosome 17	13.4	0.9	1.0	0.1
Chromosome 18	14.7	0.6	0.7	0.0

The presented cutoffs were determined by evaluation of non-disomy in 15 nonmalignant control effusions, and reflect the mean+3 s.d. percentage of aneusomic cells within each of four FISH signal categories (see Fiegl *et al*, 2000).

) were derived from the analysis of a somewhat limited number of 15 control effusions, whereas the slightly more stringent cutoffs used in series 2 were derived from 66 control effusions; we concluded that the specificity of FISH evaluation was at least 97% (Fiegl *et al*, 2004).

All test effusion specimens were routinely evaluated by cytology and classified as being benign/reactive or malignant using generally accepted criteria (Koss, 1992). Malignancy was also assumed when only a few suspicious cells (single or clustered) were present (Sears and Hajdu, 1987). Cytologic evaluation in all 81 control effusions was positive for malignancy in four cases; thus, the resulting specificity for cytology was 95%.

### Statistical analysis

McNemar's test was used to compare the two different analytical methods within the one given sample cohort of 358 effusions (Dwyer, 1991). SSPS software was used for calculations.

## RESULTS

### Patients’ characteristics

In total, 358 effusions were derived from 301 patients with histologically proven tumour disease. The most frequent underlying tumour entities were breast, lung, pancreatic, ovarian, and hepatocellular cancer (Table 1). Included in this series were also a few patients who suffered previously from a tumour and developed an effusion of unknown origin later in life. Effusion development was the primary manifestation of malignancy in 46 out of 301 patients (15.3%). A malignant effusion appeared as the first disease manifestation in 10 out of 26 cases with ovarian cancer (38.4%) and in 21 out of 60 cases (35%) with lung cancer. Effusion was the first and frequently the single sign of relapse in 44 out of 301 patients (14.6%). This was most often noted in patients with breast cancer (24 out of 67, 35.8%). Detailed frequencies of effusion as the first sign of disease or relapse according to tumour entities are listed in Table 1.

### Fluorescence *in situ* hybridisation for detection of tumour-associated aneuploidy

**Figure 1:**
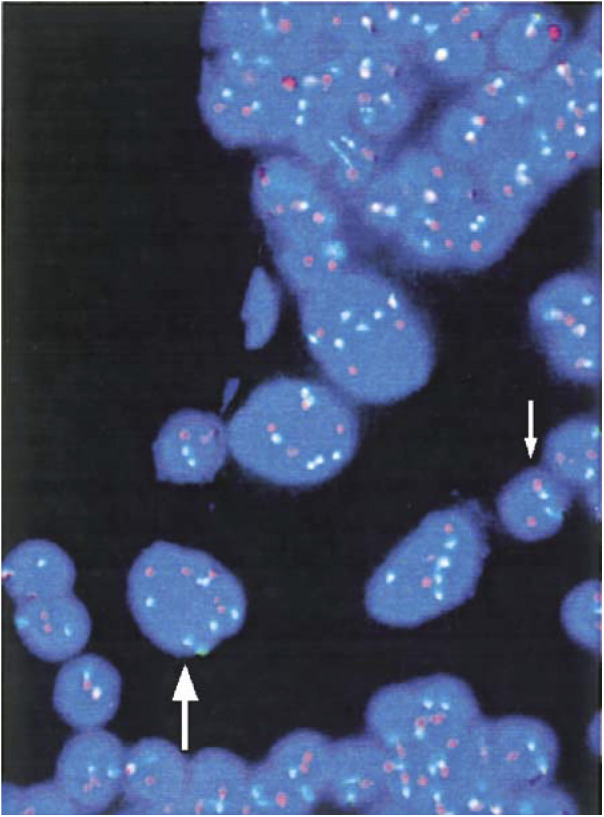
Detection of numerical chromosomal anomalies in pleural effusion cells from a patient with advanced non-small-cell lung cancer. Within a dense reactive cellular background, at least four tumour cells with marked aneuploidy can be recognised. A tumour cell with six copies of chromosome 11 (green) and four copies of chromosome 17 (orange) is marked by the large arrow. As internal control, the leukocyte (small arrow) exhibits a 2/2 signal pattern.

Fluorescence *in situ* hybridisation analysis was performed in single-colour FISH evaluation when aneusomy was present above a cutoff value, unambiguously discriminating tumour-associated aneusomy from background ‘physiological’ aneusomy. When aneusomic cells were rare, evaluation in dual-colour FISH evaluation was performed aiming at discriminating polyploidy from aneuploidy originating from tumour cells (Figure 1

). The data presented in this paper represent two compiled series of effusions analysed in two different laboratories. Slightly different cutoffs for the diagnosis of true malignancy by single- or dual-colour FISH were applied (Table 2). The larger series presented here enabled us to test the diagnostic utility of FISH in different tumour entities. Fluorescence *in situ* hybridisation analysis was diagnostic for malignant cells in 193 out of 358 effusions (53.9%), whereas cytologic evaluation was positive in 159 out of 358 (44.4%). This difference in favour of FISH was statistically significant (*P*=0.0001; McNemar's test). Taking the positive results of cytology and FISH together (cyto&FISH), the overall diagnostic yield was 60.9% (malignancy in 218 out of 358 effusions). Next, the contribution of FISH to detect malignancy in different tumour entities and tumour categories was analysed, and the results are summarised in Table 4

**Table 4:** Diagnostic sensitivities of cytology, FISH, and the combination of both (cyto&FISH) in the whole series of effusions and major tumour entities (with ⩾10 effusions per tumour entity available)

**Tumour**	**Effusion (*n*)**	**Sensitivity cytology (%)**	**Sensitivity FISH (%)**	***P***	**Sensitivity Cyto&FISH (%)**	***P***
All	358	44.4	53.9	0.0001	60.9	0.0001
*Gynaecologic*^a^	147	54.4	63.9	0.018	72.1	0.0001
Breast	104	50	63.1	0.031	71.2	0.0001
Ovarian	29	65.5	72.4	0.727	82.75	0.063
Endometrial	11	54.4	63.6	1	63.6	1
*Gastrointestinal*^b^	87	28.7	36.8	0.118	41.4	0.001
Pancreatic	34	38.2	52.9	0.125	55.9	0.031
Hepatoma	17	0	5.9	1	5.9	1
Gastric	14	50	64.3	0.625	71.4	0.25
Colorectal	11	18.2	18.2	1	27.3	1
Lung	71	54.9	71.8	0.008	76.1	0.0001
UPC^c^	17	35.3	47.1	0.687	58.8	0.125
Haematological^d^	10	50	30	0.5	50	1

The *P*-values indicate significance levels for the comparison of FISH *vs* cytology (left column) and of cyto&FISH *vs* cytology (right column).

a Gynaecologic tumours: breast (*n*=104), ovarian (29), endometrial (11), and cervical carcinoma (3).

b Gastrointestinal tumours: pancreatic (34), hepatocellular (17), gastric (14), colorectal (11), cholangiocellular (9), and oesophageal carcinoma (2).

c UPC, unknown primary cancer.

d Haematological malignancies: non-Hodgkin's lymphoma (5), chronic myelogeneous leukaemia (2), Hodgkin's lymphoma (2), idiopathic myelofibrosis (1).

. Briefly, FISH was significantly superior as compared to cytology in effusions from breast carcinoma, lung carcinoma, and gynaecological malignancies as a whole category. When the sensitivity of overall cyto&FISH was compared to that of cytology alone, the combined diagnostic workup yielded a significantly superior sensitivity for effusions from pancreatic carcinoma and all gastrointestinal tumours together.

In all, 17 effusions originated from patients who formerly suffered from malignancy but were free from manifest malignant disease at the time of puncture or thereafter. These patients had had hepatoma (*n*=8), breast cancer (*n*=3), renal, endometrial, cholangiocellular, bronchial carcinoma (*n*=1 each), carcinoma of the cervix uteri (*n*=1), and chronic myelogeneous leukaemia (*n*=1). None of these effusions was classified as malignant by cytology or FISH; thus, they were viewed as of reactive cause.

### Cytology and FISH evaluation in transudates

In the following section, the sensitivity of cytology, FISH, and the combined evaluation was compared between exsudates and transudates. Results are presented in Table 5

**Table 5:** Diagnostic sensitivity of cytology and FISH (and their combination) in transudates^a^

		**Number of transudates positive for tumour cells (%)**		
		**Cytology**	**FISH**	**Cyto&FISH**
Definition transudate	Protein <2.5 g dl^−1^ (*n*=70)	13 (18.6)	21 (30)	26 (37.1)
	Effusion/serum-protein gradient <0.5 (*n*=60)	21 (35)	24 (40)	30 (50)

a Two different common definitions, as explained in the text.

. Two different definitions to discriminate exsudates from transudates were applied: a transudate was defined by (i) a protein concentration of <2.5 g dl^−1^ (Runyon *et al*, 1992), (ii) an effusion/serum protein gradient of <0.5 (Light, 2002).

In all, 70 of the 243 effusions for which the protein concentration was available were diagnosed as transudates due to a protein concentration <2.5 g dl^−1^; in these samples, cytology, FISH, and overall cyto&FISH were diagnostic for malignancy in 18.6, 30, and 37.1%, respectively. From 100 effusion samples, protein levels from matched serum specimens were available. Out of these 100 effusions, 60 were transudates, with a protein concentration as determined by effusion protein/serum protein quotient of <0.5; in these, cytology, FISH, and cyto&FISH were diagnostic for malignancy in 35, 40, and 50%, respectively.

## DISCUSSION

In this study of patients with tumour-associated effusions, it was possible to determine the percentage of cases in whom the appearance of effusion was the first sign of primary disease or the first sign of relapse (Table 1). In the literature, information on the frequencies of effusion as the first sign of malignant disease or relapse is sparse. In our patients with invasive breast cancer, effusion was the first sign of relapse in 36% of cases. This finding is in line with results from a previously reported series (Raju and Kardinal, 1981). Of clinical relevance, the strong association between ‘new effusion of unclear origin’ and a potentially underlying tumour disease may urge performing further diagnostic steps, including analysis of effusions with molecular tools (see below).

To the best of our knowledge, this is the largest series of effusions in which conventional cytology and a molecular method such as FISH were compared for the detection of malignancy. We show that FISH analysis for the detection of tumour-associated aneuploidy is significantly more sensitive than routine cytology. In control effusions collected from patients with nonmalignant diseases, cutoffs were set for discriminating background signals from true aneuploidy, indicating the presence of tumour cells. With this reference, a sensitivity of at least 97% was achieved for FISH analysis. The diagnostic benefit of FISH was most evident in breast and lung cancer and in the group of gynaecologic malignancies. Cytology remains certainly the gold standard for the routine analysis of effusions. However, taking the positive results of cytology and FISH together (cyto&FISH), the combination of both methods brought the highest diagnostic yield (Table 4). Overall, cyto&FISH was clearly superior to cytology alone in breast, lung, and pancreatic cancer and in the categories of gynaecologic and gastrointestinal tumours. There was also a trend favouring cyto&FISH in ovarian carcinoma. When compared to cytology, FISH analysis is often complementarily diagnostic and should be used as second diagnostic step in certain cases with negative or ambiguous cytology. This may be of clinical practice in patients with known or suspected cancer. We do not propose FISH or other laborious molecular techniques as first-line diagnostic tools in all effusion samples submitted to analysis, due to a low pre-test probability of detecting malignant cells in many cases and costs.

Transudates are considered to be tumour cell contaminated in ≤5% of cases (Ashchi *et al*, 1998). Notably, cytology and FISH enabled us to detect tumour cells even in a significant proportion of transudates as defined by protein concentration and effusion/serum protein ratio. There has been intensive debate in the literature over whether or not effusions classified as transudates need any further diagnostic evaluation (Ashchi *et al*, 1998; Castro *et al*, 1998; Moltyaner *et al*, 2000). Our data support the view that, as is the case with exsudates, cytologic and molecular evaluation is warranted in transudates as well.

Fluorescence *in situ* hybridisation analysis represents only one of the molecular examinations capable of detecting malignant cells dispersed in effusions. Others are reverse-transcriptase–polymerase chain reaction and single-nucleotide polymorphism (SNP) analysis (Yang *et al*, 1998; Chang *et al*, 2002; Grünewald *et al*, 2002). The big advantage of FISH is the direct visualisation of tumour cells exhibiting numeric chromosomal aberrations (Figure 1). The diagnostic power of FISH may be further improved in two ways: first, if the primary tumour is FISH-genotyped with a panel of FISH probes for different chromosomes as part of the initial diagnostic work-up, the probe(s) best suited to indicate tumour-associated aneusomy in an individual case can be chosen to sensitively detect tumour cell involvement in effusions taken subsequently. With this procedure, we and others were able to show by FISH that primary tumours and synchronous or metachronous locoregional or distant metastases exhibit nearly identical patterns of numeric chromosomal aberrations (Simpson *et al*, 1996; Fiegl *et al*, 2000; Fehm *et al*, 2002). Second, enrichment steps like flow cytometry or immunobead selection can be applied prior to FISH analysis in order to deplete the reactive cellular background (Feuring-Buske *et al*, 1999; Sakaguchi *et al*, 1999).

Diagnosis of malignant cells in effusions or other body fluids such as lavages, urine, and cerebrospinal fluid is essential for adequate staging and prognostic evaluation. For example, in lung cancer the absence or presence of malignant cells in pleural effusions discriminates limited (e.g., T1) from locally advanced stages (e.g., T4), with very different consequences for therapeutic management. It could be decisive, before a planned resection of lung cancer, to analyse by cytology and FISH an accompanying effusion or a pleural lavage (Buhr *et al*, 1997). Improved detection of pleural metastasis by ‘molecular upstaging’ could prevent patients with more extensive disease from undergoing surgery. We demonstrated in this study that FISH can be most useful for staging procedures in patients with cytologically negative or unclear effusions. However, conventional cytology in effusions is to date the only method accepted in the classical UICC/AJCC TNM classification (Hermanek *et al*, 1999). Confirmatory prospective studies are required to demonstrate the clinical benefit of molecular methods to detect disseminated tumour cells in terms of disease outcome. Based on our experience, we believe that molecular diagnostics such as FISH will become indispensable in initial diagnosis for the diagnostic workup of primary tumour tissue and other materials (e.g., pleural lavage, bone marrow), and for restaging during the course of malignant disease.
